# Quality Assessment of Ground Coffee Samples from Greek Market Using Various Instrumental Analytical Methods, In Silico Studies and Chemometrics

**DOI:** 10.3390/antiox12061184

**Published:** 2023-05-30

**Authors:** Thalia Tsiaka, Eftichia Kritsi, Sotirios M. Bratakos, Georgios Sotiroudis, Panagiota Petridi, Ioanna Savva, Paris Christodoulou, Irini F. Strati, Panagiotis Zoumpoulakis, Dionisis Cavouras, Vassilia J. Sinanoglou

**Affiliations:** 1 Laboratory of Chemistry, Analysis & Design of Food Processes, Department of Food Science and Technology, University of West Attica, Agiou Spyridonos, 12243 Egaleo, Greece; tsiakath@uniwa.gr (T.T.); sbrat@uniwa.gr (S.M.B.); fst18684059@uniwa.gr (P.P.); ft06083@uniwa.gr (I.S.); estrati@uniwa.gr (I.F.S.); pzoump@uniwa.gr (P.Z.); 2 Institute of Chemical Biology, National Hellenic Research Foundation, 48 Vas. Constantinou Ave., 11635 Athens, Greece; gsotir@eie.gr (G.S.); pchristodoulou@eie.gr (P.C.); 3 Department of Biomedical Engineering, University of West Attica, Agiou Spyridonos, 12243 Egaleo, Greece; cavouras@uniwa.gr

**Keywords:** coffee, color parameters, total phenolics, antioxidant and antiradical activity, attenuated total reflectance Fourier transform infrared spectroscopy (ATR-FTIR), liquid chromatography-mass spectrometry (LC-MS/MS), molecular docking, discriminant analysis

## Abstract

Coffee is one of the most widely consumed beverages worldwide due to its sensory and potential health-related properties. In the present comparative study, a preparation known as Greek or Turkish coffee, made with different types/varieties of coffee, has been investigated for its physicochemical attributes (i.e., color), antioxidant/antiradical properties, phytochemical profile, and potential biological activities by combining high-throughput analytical techniques, such as infrared spectroscopy (ATR-FTIR), liquid chromatography-tandem mass spectrometry (LC-MS/MS), and in silico methodologies. The results of the current study revealed that roasting degree emerged as the most critical factor affecting these parameters. In particular, the L* color parameter and total phenolic content were higher in light-roasted coffees, while decaffeinated coffees contained more phenolics. The ATR-FTIR pinpointed caffeine, chlorogenic acid, diterpenes, and quinic esters as characteristic compounds in the studied coffees, while the LC-MS/MS analysis elucidated various tentative phytochemicals (i.e., phenolic acids, diterpenes, hydroxycinnamate, and fatty acids derivatives). Among them, chlorogenic and coumaric acids showed promising activity against human acetylcholinesterase and alpha-glucosidase enzymes based on molecular docking studies. Therefore, the outcomes of the current study provide a comprehensive overview of this kind of coffee preparation in terms of color parameters, antioxidant, antiradical and phytochemical profiling, as well as its putative bioactivity.

## 1. Introduction

Coffee is one of the most popular beverages worldwide, enjoyed by millions of people for its rich flavor, intense aroma, and stimulating effects [1]. According to a report by the International Coffee Organization, in 2020, the global population consumed approximately 167.1 million 60 kg bags of coffee [2], with the top three coffee-consuming countries including the United States, Brazil, and Germany (https://www.ico.org/ (accessed on 30 April 2023)). The coffee market is a dynamic and ever-evolving landscape, driven by the constant quest for innovation and the pursuit of consumer satisfaction. The significant role that coffee plays in the daily lives of millions of people worldwide is highlighted by the fact that the global coffee market is projected to grow at a compound annual growth rate (CAGR) of 4.65% between 2018 and 2028 (https://www.mordorintelligence.com/industry-reports/coffee-market (accessed on 30 April 2023)).

There are two main species of coffee beans that are widely cultivated for commercial consumption: *Coffea arabica* L. (Arabica coffee) and *Coffea canephora* L. (Robusta coffee) [3]. Arabica coffee is widely considered to be the superior species for its complex flavor profile and aroma, while Robusta coffee is known for its hardiness and disease resistance [4]. Arabica coffee beans impart a smoother, more nuanced flavor profile with notes of fruit, chocolate, and nuts, while Robusta coffee beans provide a more bitter and earthy taste with a higher caffeine content [5]. Among the different types of coffee preparations, “Turkish” or “Greek” coffee, which is popular in the Mediterranean region, is made by boiling finely ground roasted coffee beans inside a bronze pot known as “cezve” or “briki”.

The main chemical composition of coffee contains a wide range of compounds, comprising carbohydrates, lipids, amino acids, minerals, and various bioactive compounds [6,7]. The latter can be divided into several categories, including alkaloids, diterpenes, and phenolic compounds. Caffeine and trigonelline are two important alkaloids in coffee that contribute to its unique flavor and present potential health benefits [8,9]. Apart from the stimulation effect, caffeine, the most abundant compound in coffee, has been observed to decrease the risk of Parkinson’s and Alzheimer’s diseases and certain types of cancer [10,11]. Trigonelline, a lesser-known coffee compound, provides potential health benefits, including a reduction in the risk of chronic diseases such as type 2 diabetes and cardiovascular disease [12,13]. Cafestol and kahweol, two diterpenes of coffee, are associated with potential anti-cancer properties [14]. Additionally, cinnamic acids, cinnamaldehydes, and proanthocyanidins are among the numerous chemical constituents which impart a diverse range of properties to coffee, including antioxidant, anti-inflammatory [15], anti-diabetic, anti-cancer, cardio-protective, and antimicrobial effects [16].

Coffee consumption has also been linked with diverse potential health benefits attributed mainly to coffee’s phenolic compounds [15,17,18]. Phenolics, such as chlorogenic, caffeic, ferulic, *p*-coumaric, *p*-hydroxybenzoic, vanillic, and quinic acids, are available in abundant quantities in coffee [16]. Chlorogenic acid, the most abundant phenolic compound in coffee, possesses antioxidant, anti-inflammatory, neuro-protective, anti-viral, and anti-cancer activities [19]. Caffeic and ferulic acids are also potent antioxidants that help shield the body against oxidative stress and inflammation [16]. Quinic acid indicates antimicrobial activity and may play a role in protecting the body against certain bacterial infections [20,21]. However, extensive research is needed to fully understand the mechanisms underlying these effects and to determine the optimal levels of phenolic compounds to maximize health benefits [22]. Several studies suggested that the total phenolic content of coffee may vary depending on the type of beans and the brewing method used, with darker roasts typically displaying lower phenolic content compared to lighter roasts [23,24,25].

In the present study, different types/varieties of coffees were prepared using a method known as Greek or Turkish and investigated for their physicochemical attributes (i.e., color), their antioxidant/antiradical properties, phytochemical profile, and potential bioactivities by combining high-throughput analytical techniques, such as ATR-FTIR, LC-MS/MS, and in silico methodologies. To our knowledge, there is scarce information regarding the analysis of this specific type of coffee (Greek or Turkish coffee) using an integrated methodology, which includes physicochemical attributes, discriminant analysis, high-throughput analytical techniques, and in silico studies. In particular, the objective of the study was to (a) evaluate the quality of different coffee samples by determining their color attributes, (b) elucidate health-associated phytoconstituents by assessing the phytochemical fingerprint of coffee samples and their antioxidant/antiradical activities, and (c) understand the molecular mechanisms of coffee and its interactions with human health by implementing in silico techniques on two pharmacological targets related to diabetes and Alzheimer’s disease. 

## 2. Materials and Methods

### 2.1. Sample Collection

Fifty-seven samples of ground coffee were obtained from various Greek coffee companies. The categories, geographical origin, and related information of the ground coffee samples provided by the suppliers are presented in Table 1. The suppliers also provided information about the roasting conditions. Regarding the light, medium, and dark roasted coffee, the coffee bean’s internal temperature did not exceed 205 °C, 225 °C, and 245 °C, respectively, and the roasting time ranged between 12 and 18 min. All coffee samples were stored at 15 °C in airtight packaging in the dark until further analysis. 

### 2.2. Preparation of Brewed Coffee Beverages

Brewed coffee beverages were prepared by adding approximately 3 g of ground coffee to 50 mL of water using a coffee pot. Boiling lasted thirty-five seconds and stopped when foam formed on the brew surface. The brewed beverages were stored in closed flasks for 1 h in a refrigerator (4 °C). Then, after the dregs were separated from each coffee, the supernatants were collected and stored at 4 °C for further analysis. The preparation was repeated thrice for each coffee sample. 

### 2.3. Color Assessment

The color of ground coffee samples was measured using a tristimulus chromatometer (model CR-400, Minolta, Tokyo, Japan) calibrated with a standard white plate (L*: 97.83, a*: −0.45, b*: +1.88). The color results were expressed by the parameters L* (lightness), a* (redness/greenness), b* (yellowness/blueness), and h* (hue angle in degrees).

### 2.4. Attenuated Total Reflectance—Fourier Transform Infrared Spectroscopy (ATR-FTIR)

The FTIR spectrum was recorded at room temperature using attenuated total reflectance (ATR). Samples of ground coffee were loaded in an FTIR spectrometer (Shimadzu, IRAffinity-1S FTIR Spectrometer, Kyoto, Japan). The ATR reference was set at 3284.77 cm^−1^. The samples and the background spectra were obtained from 4000 to 499 cm^−1^, and the average of 20 scans at a resolution of 4 cm^−1^ was recorded. The FTIR spectra were exposed to ATR correction, normalization, and smoothing using the Savitzky–Golay method. Data processing and analysis were conducted using LabSolutions IR software (version 2.21, Shimadzu, IRAffinity-1S FTIR Spectrometer, Kyoto, Japan).

### 2.5. Spectrophotometric Assays

Measurements were performed in triplicate with a Spectro 23 Digital Spectrophotometer (Labomed, Inc., Los Angeles, CA, USA). The total phenolic content (TPC) was determined according to the modified method of the Folin–Ciocalteu assay [26]. Absorbance was measured at 750 nm. The results were expressed as mg of gallic acid equivalents (GAE) per 100 mL of coffee beverage, using standard solutions with a concentration range of 20–500 mg·L^−1^ gallic acid. The antiradical activity of ABTS^●+^ radical was determined using the method of Lantzouraki et al. [27]. Measurements were performed at 734 nm. Antiradical activity of samples was expressed as mg of Trolox Equivalents (TE) per 100 mL of coffee beverage, using standard solutions with a concentration range of 0.20–1.5 mM. The Ferric Reducing Antioxidant Power (FRAP) assay was determined according to the method of Lantzouraki et al. [28]. Absorbance was measured at 595 nm. Antioxidant activity was expressed as mg of Fe(II) Equivalents per 100 mL of coffee beverage, using standard solutions with a concentration range of 600–2000 uM of FeSO_4_·7H_2_O. 

### 2.6. Discriminant Analysis

For discriminating between the five different coffee categories, the color parameters, the spectrophotometric results, and the FTIR spectra intensities of all coffee samples were used and subjected to machine learning methods. Classifier algorithms from the scikit-learn library of the Python programming language (https://scikit-learn.org/ (accessed on 30 April 2023)) were employed. The accuracy of each classification algorithm was tested by assigning each coffee type to the correct category based on the above-mentioned results. This was accomplished by forming a different combination of the measured features each time, normalizing each feature to zero mean and unit standard deviation, and reducing the features–combination dimensionality by compacting the features into two principal component analysis components, PC1 and PC2. The accuracy of coffee-type classification using the K-fold evaluation method was tested. According to the K-fold method, the dataset was randomly split into 3 subsets (K = 3 in K-fold), 2 subsets were used to design the classification system, and 1 subset was used to evaluate its accuracy. That process continued by excluding a different subset each time until all subsets were used for evaluation. The K-fold cycle was repeated 10 times, and the average classification accuracy was calculated. The K-fold method was performed using the scikit-learn library’s RepeatedKFold method. The whole process (system design and precision evaluation) was repeated for different measured feature combinations and different classification algorithms. The classifiers used were Nearest Centroid, K-Nearest Neighbor, Naïve Bayesian, Logistic Regression, Linear Discriminant Analysis, Perceptron, Multi-Layer Perceptron, Random Forest, Classification and Regression Decision-Tree, and Support Vector Machines. In the end, we were able to determine the best classifier which for a particular features–combination would reveal the highest classification accuracy for the different coffee categories. For the data of the present study, the best-performing classifier was the Classification and Regression Decision-Tree (CART). Classification results were displayed using two-dimensional scatter plots of the PC1 and PC2 components. 

### 2.7. Statistical Analysis

Each physicochemical measurement was carried out in three replicates in order to record the average values and standard deviations. Results of color parameters, spectrophotometric assays, and ATR-FTIR spectra interpretation were analyzed using a significance level of *p* < 0.05 with one-way ANOVA and post hoc analysis. These calculations were carried out using SPSS (IBM SPSS Statistics. version 29.0. Chicago, IL, USA) for Windows.

### 2.8. Phytochemical Profile Using Data Dependent LC-ESI(−)-MS/MS Analysis

For the LC-MS/MS analysis, 1 mL of each coffee preparation was lyophilized, and the dry residues were diluted in 1 mL of methanol +0.1% *v*/*v* formic acid. All solvents used for the analysis were of LC-MS grade. In particular, methanol, water, acetonitrile, and formic acid were purchased from Merck KGaA (Darmstadt, Germany), Thermo Fischer Scientific (Waltham, MA, USA), Chem-Lab (Zedelgem, Belgium), and LGC Promochem (Teddington, UK), respectively.

The used LC system was an Agilent 1200 HPLC system (Agilent Technologies, Santa Clara, CA, USA), which included an Agilent Eclipse Plus C-18 reversed-phase column (50 mm × 2.1 mm inner diameter, 3.5 µm particle size) and an RRLC in-line filter kit (2.1 mm, 0.2 µm filter) (Agilent Technologies, Santa Clara, CA, USA). The mobile phase consisted of water −0.2% *v*/*v* formic acid (Solvent A) and acetonitrile −0.1% *v*/*v* formic acid (Solvent B). The MS system contained a 3200 Q TRAP triple-quadrupole linear ion trap mass spectrometer (Sciex, Framingham, MA, USA). MS acquisition was performed with an electrospray (ESI) ionization source in the negative mode, which is the preferred ionization mode for the phenolic compounds. Moreover, the data-dependent MS/MS analysis was conducted by applying information-dependent acquisition (IDA)-triggered MS/MS scans (EPI—enhanced product ion scans) [29]. The mass error was 0.1 Da in MS and 0.5 Da in MS/MS. All the technical and instrumentation-related information regarding the LC-MS/MS method used for the elucidation of the phenolic compounds of coffee samples are described in detail in previous publications of our group [29,30]. 

The elucidation of phenolic compounds was carried out using a library developed in-house, which contained phenolic acids and flavonoids [29]. Furthermore, additional characteristic mass fragments, apart from those corresponding to the phenolic compounds of the in-house library, were also identified according to literature data [31]. The contents of the annotated compounds in the coffee sample were expressed based on the normalized relative intensities of the precursor ions after identifying them through their characteristic fragmentation pattern and their RTs (in the case of phenolic acids which were included in the in-house library). All LC-MS/MS spectra were processed using Analyst software (version 1.4.2) (Sciex, Framingham, MA, USA). The statistical analysis of LC-MS/MS results was performed with an ANOVA General Linear Model test at a confidence level of 95% using the Minitab suite (trial version 20, Minitab LLC, State College, PA, USA).

### 2.9. In Silico Inhibitory Activity of Principal Coffee Phenolic Compounds against Acetylcholinesterase and α-Glucosidase Enzymes

Molecular docking studies were employed to explore the potential interaction pattern between characteristic identified phenolic acids of examined coffee samples and targets related to the anti-diabetic and anti-Alzheimer’s activity. For the present scope, the crystal structures of human acetylcholinesterase in complex with tacrine (PDB ID: 7XN1) and of human alpha-glucosidase complexed with acarbose (PBD ID: 2QMJ) were retrieved from the Protein Data Bank (https://www.rcsb.org, accessed on 10 April 2023) and were prepared by applying the Protein Preparation Wizard [32]. In particular, in the selected crystal structures, all missing residues and hydrogen atoms were added, bond orders were assigned, and the crystal structures were minimized using the OPLS3 force field. Simultaneously, the phenolic acids contained in coffee samples were prepared at pH = 7.5 ± 0.5 by performing LigPrep [33]. 

Finally, all compounds were subjected to molecular docking simulations by implementing the Glide application [34] in standard precision (SP) and in extra precision (XP) mode of the Maestro interface [35]. For both crystal structures, a grid box with dimensions 10 × 10 × 10 Å was generated, and the maximum number of poses was defined as equal to 10. 

## 3. Results 

### 3.1. Color Parameters of Coffee Sample Categories

Color affects consumer acceptance since it is related to the appearance of the food. Color parameters are especially important indicators of coffee taste and aroma and verify the roasting quality [36]. The results of color measurements are given in Table 2.

Regarding the results among light, medium, and dark roast blends, it seems that the roast degree affected color parameters, resulting in a significant (*p* < 0.05) reduction in lightness (L*), redness (a*), yellowness (b*), and hue (h), from light to intense roasting. Specifically, with the increase in roasting degree and according to the hue angle values, the coffee samples’ color is transitioning from ochre orange to rust orange and spice orange. Moreover, decaffeinated and aroma blends presented intermediate values in color parameters, ranking between lightly and moderately roasted blends. 

### 3.2. Spectrophotometric Assays of Brewed Coffee Beverages

The brewed beverages of the coffee samples were analyzed spectrophotometrically in order to determine their total phenolic content with the Folin–Ciocalteu method, antiradical activity with the ABTS radical scavenging method, and antioxidant activity with the FRAP method. Table 3 lists the overall results per coffee sample category in terms of their statistical evaluation.

The average values of TPC in brewed beverages of the coffee samples ranged from 77.51 to 155.69 mg/100 mL of coffee beverage. Mean values for antiradical and antioxidant activity ranged from 278.13 to 342.93 mg TE/100 mL of coffee beverage and from 1301.79 to 1548.96 mg Fe (II)E/100 mL of coffee beverage, respectively. The lowest (*p* < 0.05) TPC, antiradical, and antioxidant activity were found when the dark roast blend was used for the preparation of brewed beverages. This finding indicates that the roasting degree significantly affects the total phenolic content and antioxidant activity of the brewed coffee, in agreement with the results of previous studies [37,38,39]. Regarding the aroma blends, which were enriched with spices, flavoring, or aromatic plants, they presented the highest (*p* < 0.05) total phenolic content and among the highest antioxidant-antiradical activity. 

Furthermore, quite high positive correlations were found among TPC and antiradical activity (0.649, *p* < 0.01), TPC and antioxidant activity (0.746, *p* < 0.01), plus antiradical and antioxidant activity (0.684, *p* < 0.01). Hence, it seems that phenolic constituents of brewed beverages of the coffee samples considerably define their antiradical and antioxidant capacity. 

### 3.3. Interpretation of ATR-FTIR Spectra

The coffee samples were analyzed using Attenuated Total Reflection-Fourier Transform Infrared (ATR-FTIR) spectroscopy. Infrared spectra were obtained in the wavenumber range from 4000 to 499 cm^−1^. The spectra evaluation of coffee samples, which was achieved according to the characteristic absorption bands listed in Table 4, revealed twenty-one different bands. The ATR-FTIR spectra evaluation was based on the comparative study of the relative intensities of FTIR spectra bands that arose after ATR correction, smoothing, and normalization. The most interesting outcomes are mentioned below. 

The intensities at 2922, 2855, 1640–1660, 1242–1218, and 1028 cm^−1^, which were associated with the presence of caffeine [40,41,42,43], exhibited a significant or dramatic (*p* < 0.05) decrease in decaffeinated blends compared to caffeinated ones. The intensities at 1381–1376, 1161–1153, and 1053 cm^−1^, which were related to the presence of chlorogenic acid [44,45,46], displayed insignificant (*p* > 0.05) variations among the studied blends, except for the aromatic blends, which exhibited increased (*p* < 0.05) intensities at 1381–1376 and 1161–1153 cm^−1^. Moreover, the absorption detected at 1603 cm^−1^ [47], which probably corresponds to the conjugated C=C stretch vibration found in diterpenes such as cafestol and kahweol, showed a significant (*p* < 0.05) decrease from light to dark roast blends, without being affected by the decaffeination process. Furthermore, the intensity at 1743 cm^−1^, which is related to the presence of quinic acid esters [48] formed during coffee roasting via chlorogenic acid hydrolysis [49], was found significantly (*p* < 0.05) increased in aroma blends, without being affected by the decaffeination process. Interestingly, the intensities at 3640–3530 and 1600–1500 cm^−1^, which were associated with the presence of aromatic compounds, especially phenolics [47], presented their highest (*p* < 0.05) values for decaffeinated blends without being affected by the roasting process.

Additionally, the intensities at 860–800 and 770–735 cm^−1^, which are related to the presence of disubstituted aromatics, as well as the absorbance at 3130–3010 cm^−1^ of C-H stretching vibration in the aromatic ring [47], revealed that the decaffeination process negatively (*p* < 0.05) affected the presence of these compounds. 

In a further step, the ratios between the intensities of the most characteristic absorption bands were calculated in order to define the blend category (Table 5). According to the results, when the ratios of the bands 2922:2855 cm^−1^ and 1028:1163 cm^−1^ are lower than 1.6 and 0.3, respectively, they are attributed to decaffeinated blends. Moreover, the roasting process seems to significantly reduce the ratio of the bands 2922:2855 cm^−1^. Furthermore, the ratios of the bands 1743:2922 cm^−1^ and 1743:2855 cm^−1^ showed a significant increase in both aroma and decaffeinated blends. 

### 3.4. Discriminant Analysis

**Figure 1:**
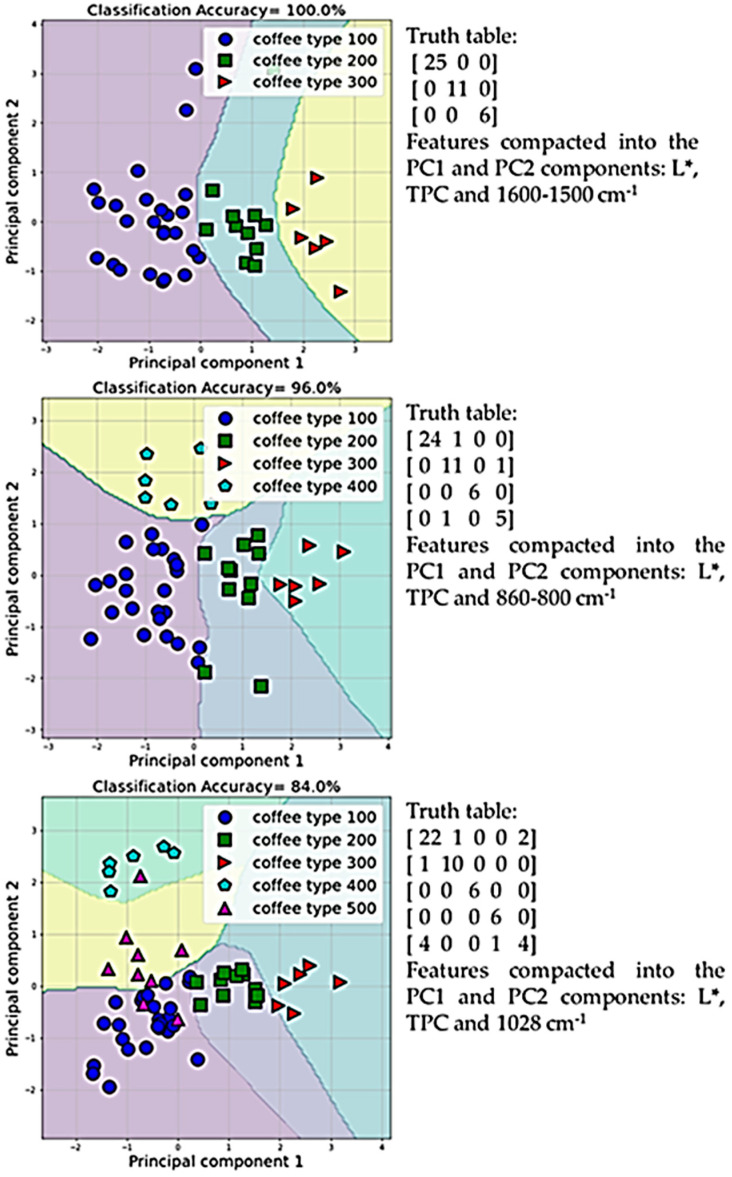
Principal component analysis scatter diagram between L*, TPC, and different FTIR bands among the studied coffee blends.

Discriminant analysis was achieved by applying machine learning algorithms to color parameters, spectrophotometric results, and FTIR spectra band intensities for defining the more appropriate feature combinations in order to confirm the discrimination of coffee sample categories. Figure 1 presents indicative PCA (Principal Component Analysis) scatter diagrams, highlighting the successful classification of coffee blend categories. The first diagram shows the optimal classification of the coffee categories of different roasting degrees, with a 100.0% overall discrimination accuracy, based on the features L* (lightness), TPC (total phenolic content), and the intensity of the FTIR band at 1600–1500 cm^−1^. The second diagram presents the best classification of all coffee categories (except aroma blends), and the third is the best classification of all coffee categories. The overall accuracy of predicting the coffee categories, except for aroma blends, was 96.0%, whereas the accuracy of all coffee categories classification was 84.0%. 

### 3.5. Metabolite Identification of Coffee Samples via LC-MS/MS

The assessment of LC-MS/MS data resulted in the annotation of 18 coffee-related phytochemicals, which pertain to the groups of phenolic acids (4 compounds), organic acids (1 compound), hydroxycinnamate esters and lactones (5 compounds), diterpenes (5 compounds), fatty acids and derivatives (2 compounds), and hydroxycinnamoyl amides (1 compound). All the identified metabolites with their RTs and fragmentation patterns are presented in Table 6.

**Figure 2:**
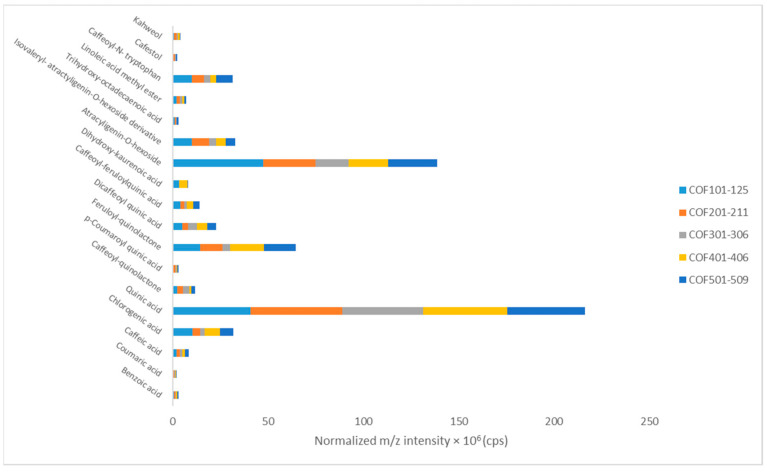
Comparison of the mean contents of detected metabolites in coffee samples.

Benzoic acid, caffeic acid, and chlorogenic acid were the phenolic acids detected in all coffee samples regardless of their roasting process, their variety, or their type (decaffeinated, flavor, origin, etc.). However, only the mean contents of chlorogenic acid showed significant differences (*p* ≤ 0.05), as they were significantly lower as the degree of roasting increased (Group 2 and 3, medium and black roasted coffees, Table 1). Quinic acid, along with caffeic acid, which are considered key coffee metabolites since they are products of chlorogenic acids reactions during roasting, presented the highest content among all the elucidated metabolites in coffee samples (Figure 2). 

As indicated in Figure 2, the roasting level and conditions also affected the mean contents of quinic and chlorogenic acid derivatives (i.e., caffeoyl-quinolactone, feruloyl-quinolactone, dicaffeoyl quinic acid, etc.), which are major markers of coffee brewing and final sensory quality [50]. Although the comparison of the mean content of these compounds did not reveal any critical differences between the sample groups (*p* > 0.05), considerable variances were reported in certain samples that belong to the same group (i.e., feruloyl-quinolactone content in sample 114 was significantly higher compared to sample 116, Table S1) [51]. The mean contents, expressed by the normalized mass intensities of each compound, and their variances are reported in Table S1 (Supplementary Data). According to our findings, the diterpene dihydroxy-kaurenoic acid was not detected in the samples with high roasting degrees (medium and dark roasted blends). Furthermore, the mean contents of the diterpenes cafestol and kahweol, which according to recent bibliographic data [52], are related to coffee variety and roasting degree, did not differ significantly (*p* > 0.05) between the sample groups (Table 1). On the other hand, the diterpene atracyligenin-O-hexoside presented significantly higher values (*p* ≤ 0.05) in light and medium roasted and in flavored coffees, while its mean content was reduced in dark roasted and decaffeinated samples. The levels of trihydroxy-octadecaenoic acid, linoleic acid methyl ester, and caffeoyl-N-tryptophan were similar in all samples (*p* > 0.05). The chromatographic peaks of selected metabolites are illustrated in Figure S1 (Supplementary Data).

### 3.6. Molecular Docking Results Evaluation

**Figure 3:**
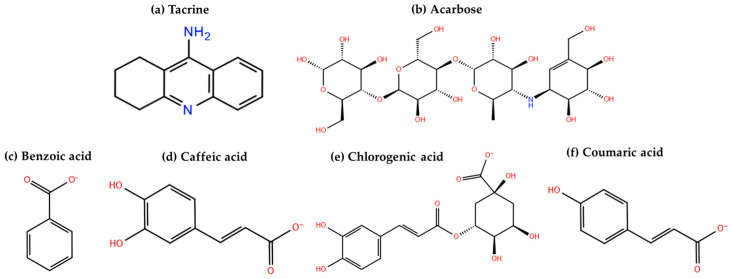
Chemical structures of (**a**) tacrine and (**b**) acarbose, the co-crystallized ligands of the examined molecular targets, and (**c**) benzoic acid, (**d**) caffeic acid, (**e**) chlorogenic acid, and (**f**) coumaric acid at pH = 7.5 ± 0.5.

The characteristic phenolic acids of coffee samples determined through the developed LC-ESI(−)-MS/MS in-house library, including benzoic acid, caffeic acid, chlorogenic acid, and coumaric acid (Figure 3), were subjected to molecular docking studies in an effort to investigate their potential inhibitory affinity against human acetylcholinesterase (PDB ID: 7XN1) and human alpha-glucosidase enzymes (PDB ID: 2QMJ). It is noted that the presence of the aforementioned phenolic compounds in coffee samples was also confirmed by recent literature data [16,18]. The molecular target selection was based on the fact that they constitute well-established anti-Alzheimer’s and anti-diabetic targets, respectively [16,53]. 

**Figure 4:**
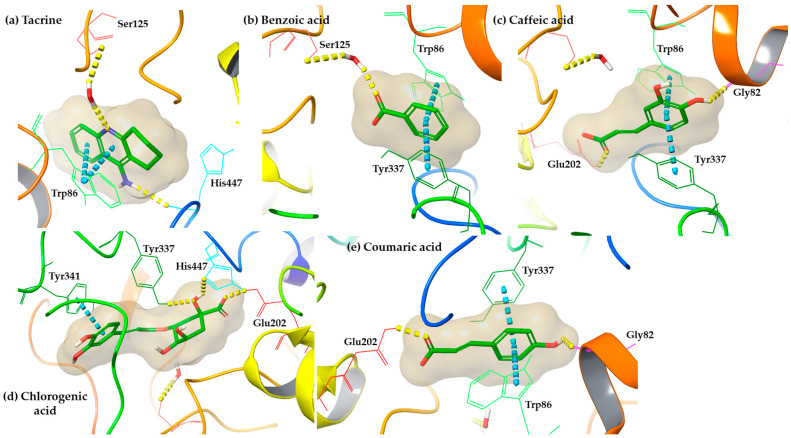
Representative binding poses of (**a**) the co-crystallized ligand, tacrine, (**b**) benzoic acid, (**c**) caffeic acid, (**d**) chlorogenic acid, and (**e**) coumaric acid, generated from molecular docking simulations into human acetylcholinesterase enzyme (PDB ID: 7XN1). The color depiction is as follows: hydrogen bonds are depicted with dashed yellow lines and π-π stacking with blue dashed lines.

Concerning the human acetylcholinesterase enzyme, all tested compounds present reasonable docking score values compared to the co-crystallized inhibitor tacrine (Table 7). However, their docking poses indicated a fruitful interaction pattern, containing similar tacrine interactions (Figure 4). Particularly, the aromatic ring of benzoic acid interacts via a pi–pi stacking with Trp86, like the co-crystallized ligand and Tyr337. Moreover, a water-bridged hydrogen bond is formed with Ser125 as the co-crystallized inhibitor. In the case of caffeic acid and coumaric acid, a similar benzoic acid interaction pattern is illustrated, containing π-π interactions with Trp86 and Tyr337. Additionally, caffeic and coumaric acid also forms hydrogen bonds with Glu202 and Gly82, which further stabilize the binding affinity; docking poses indicated the formation of hydrogen bonds with Glu202, His447, and Tyr337 and a π-π stacking with Tyr341. It is critical to note that the described interactions are in accordance with the results of a recent publication [16]. 

**Figure 5:**
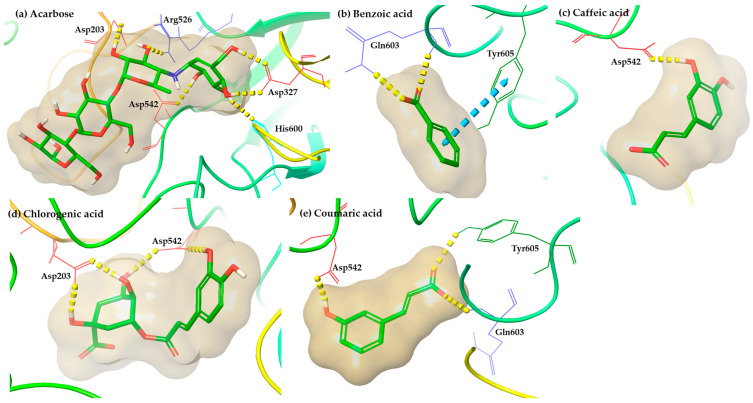
Representative binding poses of (**a**) the co-crystallized ligand, acarbose, (**b**) benzoic acid, (**c**) caffeic acid, (**d**) chlorogenic acid, and (**e**) coumaric acid, generated from molecular docking simulations into human alpha-glucosidase enzyme (PDB ID: 2QMJ). The color depiction is as follows: hydrogen bonds are depicted with dashed yellow lines and π-π stacking with blue dashed lines.

In general, molecular docking analysis results at the binding site of human alpha-glucosidase enzyme (PDB ID: 2QMJ) revealed that among the examined phenolic acids, chlorogenic and coumaric acid possess the most stable interaction pattern and may contribute to the exploration of novel alpha-glucosidase inhibitors. Chlorogenic and coumaric acid display acceptable binding energy values compared to the co-crystallized inhibitor, acarbose (Table 7), and their visual inspection pointed out a variety of interactions similar to acarbose (Figure 5). Especially, chlorogenic acid creates hydrogen bonds with Asp203 and Asp542, simulating the binding mode of acarbose. In continuation, the binding mode analysis of coumaric acid showed the formation of direct hydrogen bonds with Asp542, as acarbose, Gln603, Tyr605, and a π-π interaction with Phe575. Finally, the binding poses evaluation indicated that benzoic and caffeic acid present a reduced binding affinity into the binding site of human alpha-glucosidase. Particularly, the interaction motif of benzoic acid includes the development of a hydrogen bond with Gln603 and a π-π stacking with Tyr605, amino acids that are not participating in the binding of acarbose, and in the case of caffeic acid, only a hydrogen bond with Asp542, as acarbose, is observed (Figure 5). 

## 4. Discussion

As indicated by the determination of color parameters (Section 3.1), very strong positive correlation values were observed among L*, a*, b*, and h values of the studied coffee samples of different roasting levels (L*-a*: 0.962, L*-b*: 0.992, L*-h: 0.920, a*-b*: 0.961, a*-h: 0.899, and b*-h: 0.946, *p* < 0.01). In accordance with the literature, the roasting treatment triggers several chemical changes inside the coffee bean as a result of the Maillard reaction and caramelization, resulting in darkening products and melanoidins production and the transition of coffee color from light to dark brown [37]. It is also worth mentioning that the study of Munchow et al. [54] concluded that in roasted coffee beans, the parameters L*, b*, and h had an antagonist interaction due to an increase in the roasting intensity. Furthermore, Yeager et al. [55] reported that the increase in roasting level had a strong effect on color parameters, resulting in a significant decrease in L*, a*, and b* with a strong linear correlation among them.

Surprisingly, decaffeinated blends were found to contain among the highest total phenolic content and antioxidant–antiradical activities, suggesting that the decaffeination process does not degrade the coffee’s composition in phenolic bioactive compounds. In accordance with this finding, Hall et al. [56] reported that coffee bioactive compounds’ quantity did not differ between caffeinated and decaffeinated coffee. Generally, the results of the spectrophotometric assays are related, to a great extent, to brewing conditions (coffee-to-water ratio, brewing duration, and temperature) and brewing preparation since the beverages were boiled and not filtered. Combining the results of Folin–Ciocalteu, FRAP, and ABTS^●+^ with those of the literature, it seems that the antioxidant superiority of coffee beverages depends on the coffee roasting degree, the method and duration of the beverage brewing, the blend variety and geographic origin, the addition of flavorings, the decaffeination process, etc. [1,56,57,58,59,60].

In addition, the interpretation of ATR-FTIR spectra confirmed the presence of diterpenes cafestol and kahweol. According to literature data, diterpenes content depends on many factors, such as roasting temperatures and duration, preparation method, coffee variety, etc. [61]. It is also reported [62] that cafestol and kahweol have various pharmacological properties, such as anti-diabetic, anti-tumor, anticarcinogenic, anti-inflammatory, anti-angiogenic, antioxidant, and hepatoprotective. Furthermore, the bands related to quinic esters highlighted the effect of roasting since these esters are products of chlorogenic acid hydrolysis that occurred during this process [48,49]. As expected, aroma blends were phenolic-rich preparations [47]; however, it was quite interesting that both decaffeination and roasting processes did not affect the phenolic content of decaffeinated samples.

Regarding the discriminant analysis performed on coffee samples, it is important to mention that in all discriminations, both lightness and total phenolic content were the most determinant features. Another important observation was that aroma blends were mostly misclassified compared to the others, probably because their composition is mainly affected by the flavoring addition [63]. In conclusion, the discriminant analysis clearly exposed the effect of the roasting and decaffeination process on the physicochemical profile of the studied coffee samples.

Focusing on the LC-MS/MS results, the decrease in chlorogenic acid was anticipated as the roasting degree increased due to the decomposition of this phenolic acid upon roasting and its conversion to quinic and caffeic acids [64,65]. Moreover, factors other than roasting (i.e., cultivar, geographical origin, post-harvest process, bean maturity stage, etc.) seem to contribute to the actual content of coffee odorants’ precursors, such as quinic and chlorogenic acid derivatives, in the final coffee preparations [51]. It is also worth commenting on the effect of roasting level and coffee bean variety on the content of coffee diterpenes [52]. As observed, the outcomes of these factors were not similar for all diterpenes since, in some cases (i.e., cafestol and kahweol), their content did not change with roasting, while in other cases, intense roasting resulted in a significant decrease in certain diterpenes (i.e., dihydroxy-kaurenoic acid and atracyligenin-*O*-hexoside).

In molecular docking studies, the described interaction motif underscored the potential of caffeic acid to inhibit the human acetylcholinesterase enzyme. Moreover, in the case of coumaric acid, a common to caffeic acid interaction profile was observed, reinforcing the hypothesis that coumaric acid exhibits anti-Alzheimer activity. Among examined compounds, chlorogenic acid emerged as the most promising human acetylcholinesterase inhibitor due to its high binding affinity (docking score = 7.2 kcal mol^−^^1^) and the generation of direct hydrogen bonds and π-π interactions into human acetylcholinesterase enzyme [19]. Furthermore, chlorogenic and coumaric acid exhibited stable interaction patterns as potential alpha-glucosidase inhibitors [53]. The presented results provide insights into the potential pharmacological benefits of consuming coffee and the role of its phenolic acids in human health. However, further research is necessary to validate the effects of these compounds on human health in vivo.

## 5. Conclusions

Coffee has consolidated its position as one of the most consumed beverages worldwide due to its unique sensory characteristics and established health-promoting effects. However, the final quality of the coffee preparations depends on various factors, including coffee bean variety/cultivar, coffee origin, environmental parameters, the roasting degree, post-harvesting processes, different preparation procedures of the coffee drinks, etc. 

According to the results of the current study, which was focused on the study of Greek or Turkish coffee types, the roasting level affects various quality characteristics of the final infusions. In particular, the color and TPC values of dark-roasted coffees were downgraded compared to lighter-roasted coffees. Interestingly, decaffeinated and flavored coffees presented high phenolic content and antiradical and antioxidant activity. This finding was confirmed by ATR-FTIR spectra. The interpretation of ATR-FTIR results also revealed the higher intensities of diterpenes and quinic esters-related peaks in light roasted samples and in flavored blends, respectively. Furthermore, the ratios of the bands 1028:1163, 1743:2922, and 1743:2855 cm^−^^1^ could be recommended to differentiate decaffeinated coffees, whereas the ratio 2922:2855 cm^−^^1^ to characterize the roasting level. Furthermore, the discriminant analysis based on the color, TPC, and ATR-FTIR results showed that the coffee samples could be accurately classified based on their type (different roasting levels, decaffeinated, and coffees with flavors). Moreover, the assessment of the LC-MS/MS analysis affirmed the presence of phenolic acids, diterpenes, fatty acids, amides, and hydroxycinnamate derivatives. However, the compounds that can be potentially characterized as markers for coffee discrimination were chlorogenic acid, dihydroxy-kaurenoic acid, and atracyligenin-*O*-hexoside, while quinic acid and feruloyl-quinolactone were the metabolites with the higher contents in all samples. Moreover, the outcomes of in silico studies provided further insights into the health-related role of phenolic acids against Alzheimer’s and diabetes, as chlorogenic acid showed promising results as an inhibitor of human acetylcholinesterase enzyme and of alpha-glucosidase, in this case along with coumaric acid.

Nonetheless, the incorporation of additional samples of different types in the sample set under study, the identification of more metabolites through untargeted metabolomics, and statistical analysis suites/tools will provide further evidence concerning the validated markers of quality or classification of different coffee samples.

## Figures and Tables

**Table 1:** Presentation of ground coffee samples.

Ground Coffee Code	Ground Coffee Category	Species	Geographical Origin
101	Traditional-Blonde light roast blend	Arabica-Robusta	Brazil, Colombia, Ethiopia, India
102		Arabica-Robusta	Costa Rica, Guatemala, Colombia
103		Arabica-Robusta	Brazil, Ethiopia
104		Arabica-Robusta	Brazil, Ethiopia
105		Arabica-Robusta	Brazil, Ethiopia
106		Arabica-Robusta	Brazil, Ethiopia
107		Arabica-Robusta	Ethiopia
108		Arabica	Brazil
109		Arabica-Robusta	Brazil, India
110		Arabica-Robusta	Brazil, India
111		Arabica	Brazil, Ethiopia
112		Arabica-Robusta	Brazil, Ethiopia
113		Arabica-Robusta	Brazil, India
114		Arabica-Robusta	Brazil, India
115		Arabica	Brazil, Kenya, Colombia
116		Arabica	Brazil, Ethiopia
117		Arabica	Harar (Ethiopia)
118		Arabica	Kenya
119		Arabica	Colombia
120		Arabica	Brazil, Ethiopia
121		Arabica	Guatemala
122		Arabica	Colombia
123		Arabica	Santos (Brazil)
124		Arabica-Robusta	South America, Africa, India
125		Arabica-Robusta	South America, India
201	Medium roast blend	Arabica-Robusta	Brazil, Ethiopia
202		Arabica	Brazil, Colombia
203		Arabica-Robusta	Ethiopia
204		Arabica-Robusta	Brazil, India
205		Arabica	Brazil, Ethiopia
206		Arabica-Robusta	Brazil, India
207		Arabica	Brazil, India
208		Arabica-Robusta	Costa Rica, Guatemala, Colombia
209		Arabica	Guatemala
210		Arabica	Peru
211		Arabica	Limu (Ethiopia)
301	Dark roast blend	Arabica-Robusta	Brazil, Ethiopia
302		Arabica	Brazil, Colombia
303	Dark roast blend	Arabica-Robusta	Ethiopia
304		Arabica-Robusta	Brazil, India
305		Arabica-Robusta	Brazil, India
306		Arabica-Robusta	Costa Rica, Guatemala, Colombia
401	Decaffeinated blend	Arabica-Robusta	Brazil, Colombia, India
402		Arabica-Robusta	Ethiopia
403		Arabica-Robusta	Brazil, India
404		Arabica	Brazil, Central America
405		Arabica	Colombia
406		Arabica	Colombia
501	Aroma Blend—Mastiha	Arabica-Robusta	Brazil, India
502	Aroma Blend—Mastiha		Ethiopia
503	Aroma Blend—Mastiha		Brazil, India
504	Aroma Blend—Cardamom		Ethiopia
505	Aroma Blend—Cardamom		Brazil, India
506	Aroma Blend—Baklava		Brazil, India
507	Aroma Blend—Hazelnut		Brazil, India
508	Aroma Blend—Mastiha		Brazil, India
509	Aroma Blend—Spices		Ethiopia

**Table 2:** Comparative study of color parameters of coffee sample categories.

Coffee Samples’ Categories	Lightness (L*) *	Redness/Greenness (a*) *	Yellowness/Blueness (b*) *	Hue Angle (h) *
Traditional-Blonde light roast blends (100–125)	38.75 ± 1.48 ^a^	5.70 ± 0.71 ^a^	8.02 ± 1.73 ^a^	54.05 ± 3.88 ^a^
Medium roast blends (201–211)	34.85 ± 0.86 ^bc^	3.64 ± 0.67 ^b^	3.37 ± 1.04 ^b^	41.94 ± 3.94 ^b^
Dark roast blends (301–306)	34.16 ± 0.82 ^b^	3.13 ± 0.78 ^b^	2.41 ± 0.90 ^b^	36.34 ± 5.87 ^b^
Decaffeinated blends (401–406)	36.64 ± 1.35 ^c^	4.86 ± 0.68 ^ac^	5.84 ± 1.68 ^c^	49.48 ± 3.85 ^a^
Aroma blends (501–509)	36.47 ± 1.35 ^c^	4.67 ± 0.62 ^c^	5.67 ± 1.61 ^c^	49.63 ± 4.31 ^a^

* The results are expressed as Average (±standard deviation); ^a–c^ Different letters in the same column indicate statistically different values (*p* < 0.05).

**Table 3:** Comparative study of total phenolic content, antiradical, and antioxidant activity results of coffee sample categories.

Coffee Samples Categories	TPC (mg GAE/100 mL of Coffee Beverage) *	ABTS (mg Trolox (TE)/100 mL of Coffee Beverage) *	FRAP (mg Fe(II)/100 mL of Coffee Beverage) *
Traditional-Blonde light roast blends (100–125)	136.49 ± 13.52 ^a^	333.56 ± 22.57 ^a^	1498.49 ± 29.03 ^ac^
Medium roast blends (201–211)	117.93 ± 11.59 ^b^	328.70 ± 29.04 ^a^	1464.19 ± 46.01 ^a^
Dark roast blends (301–306)	77.51 ± 9.25 ^c^	278.13 ± 19.78 ^b^	1301.79 ± 103.00 ^b^
Decaffeinated blends (401–406)	144.23 ± 13.02 ^ad^	342.93 ± 36.70 ^a^	1548.96 ± 18.61 ^c^
Aroma blends (501–509)	155.69 ± 23.18 ^d^	322.58 ± 26.66 ^a^	1499.10 ± 33.83 ^ac^

* The results are expressed as Average (±standard deviation); ^a–d^ Different letters in the same column indicate statistically different values (*p* < 0.05).

**Table 4:** Relative intensities of ATR-FTIR spectra bands of coffee sample categories.

Functional Groups	Characteristic Absorption Bands	Traditional-Blonde Light Roast Blends (100–125) *	Medium Roast Blends (201–211) *	Dark Roast Blends (301–306) *	Decaffeinated Blends (401–406) *	Aroma Blends (501–509) *
O–H stretch in phenols	3640–3530	0.014 ± 0.003 ^a^	0.009 ± 0.002 ^a^	0.012 ± 0.003 ^a^	0.025 ± 0.007 ^b^	0.013 ± 0.003 ^a^
O–H stretch in alcohols	3500–3300	0.010 ± 0.003 ^a^	0.010 ± 0.003 ^a^	0.010 ± 0.001 ^a^	0.012 ± 0.004 ^a^	0.010 ± 0.002 ^a^
O–H stretch in water	3280	0.006 ± 0.003 ^a^	0.007 ± 0.003 ^a^	0.002 ± 0.001 ^b^	0.006 ± 0.003 ^a^	0.006 ± 0.002 ^a^
C–H stretch in aromatic ring	3130–3010	0.044 ± 0.002 ^a^	0.044 ± 0.003 ^a^	0.043 ± 0.002 ^ab^	0.040 ± 0.002 ^b^	0.042 ± 0.005 ^ab^
C–H asymmetric and symmetric stretch of CH_2_ and CH_3_ in lipids and caffeine	2922	0.679 ± 0.016 ^a^	0.688 ± 0.019 ^a^	0.679 ± 0.016 ^a^	0.538 ± 0.016 ^b^	0.691 ± 0.017 ^a^
	2855	0.375 ± 0.011 ^a^	0.381 ± 0.010 ^a^	0.383 ± 0.009 ^a^	0.344 ± 0.009 ^b^	0.380 ± 0.010 a
C=O stretch in aliphatic esters	1743	0.282 ± 0.016 ^a^	0.275 ± 0.018 ^a^	0.280 ± 0.020 ^a^	0.313 ± 0.021 ^ab^	0.328 ± 0.022 ^b^
C=O stretch in amides	1640–1660	0.031 ± 0.004 ^a^	0.025 ± 0.003 ^a^	0.026 ± 0.003 ^a^	0.012 ± 0.003 ^b^	0.026 ± 0.004 ^a^
conjugated C=C stretch	1603	0.011 ± 0.003 ^a^	0.008 ± 0.001 ^ab^	0.007 ± 0.002 ^b^	0.008 ± 0.002 ^ab^	0.010 ± 0.003 ^ab^
aromatic ring stretch	1600–1500	0.007 ± 0.007 ^a^	0.010 ± 0.007 ^ac^	0.011 ± 0.005 ^ac^	0.028 ± 0.004 ^b^	0.014 ± 0.007 ^c^
C–H scissoring bend of CH_2_	1485–1445	0.079 ± 0.019 ^ab^	0.066 ± 0.027 ^a^	0.086 ± 0.007 ^ab^	0.068 ± 0.020 ^ab^	0.093 ± 0.005 ^b^
O–H angular bend	1410–1420	0.007 ± 0.002 ^ab^	0.006 ± 0.001 ^a^	0.007 ± 0.001 ^ab^	0.009 ± 0.003 ^b^	0.008 ± 0.001 ^ab^
O–H bend in organic acids	1381–1376	0.064 ± 0.003 ^a^	0.062 ± 0.003 ^a^	0.059 ± 0.004 ^a^	0.062 ± 0.003 ^a^	0.075 ± 0.005 ^b^
C–N stretch	1242–1218	0.042 ± 0.006 ^a^	0.046 ± 0.002 ^a^	0.045 ± 0.004 ^a^	0.026 ± 0.003 ^b^	0.050 ± 0.007 ^a^
C–O stretch in organic acids	1161–1153	0.086 ± 0.003 ^a^	0.083 ± 0.003 ^ab^	0.083 ± 0.003 ^ab^	0.077 ± 0.005 ^b^	0.092 ± 0.004 ^c^
C–O bend	1053	0.018 ± 0.003 ^a^	0.021 ± 0.005 ^a^	0.025 ± 0.004 ^a^	0.021 ± 0.005 ^a^	0.022 ± 0.004 ^a^
side-chain N–CH_3_ stretch/C–O–H and C–O–C bend	1028	0.088 ± 0.009 ^a^	0.088 ± 0.006 ^a^	0.087 ± 0.007 ^a^	0.022 ± 0.002 ^b^	0.073 ± 0.011 ^c^
C–H bend in alkenes	869	0.026 ± 0.006 ^a^	0.028 ± 0.004 ^a^	0.027 ± 0.006 ^a^	0.024 ± 0.002 ^a^	0.025 ± 0.004 ^a^
C–H out-of-plane bend in para-substituted aromatics	860–800	0.052 ± 0.005 ^a^	0.052 ± 0.006 ^a^	0.051 ± 0.003 ^a^	0.039 ± 0.003 ^b^	0.049 ± 0.005 ^a^
C–H out-of-plane bend in ortho-substituted aromatics	770–735	0.015 ± 0.002 ^a^	0.014 ± 0.001 ^a^	0.013 ± 0.003 ^a^	0.010 ± 0.001 ^b^	0.015 ± 0.001 ^a^
C–H rocking bend of CH_2_	745–705	0.018 ± 0.008 ^a^	0.023 ± 0.004 ^a^	0.016 ± 0.011 ^a^	0.020 ± 0.007 ^a^	0.022 ± 0.005 ^a^

* The results are expressed as Average (±standard deviation); ^a–c^ Different letters in the same row indicate statistically different values (*p* < 0.05).

**Table 5:** Intensities ratio among ATR-FTIR spectra bands of coffee sample categories.

Intensities Ratios	Traditional-Blonde Light Roast Blend (100–125) *	Medium Roast Blend (201–211) *	Dark Roast Blend (301–306) *	Decaffeinated Blend (401–406) *	Aroma Blend (501–509) *
2922/2855	1.81 ± 0.02 ^a^	1.81 ± 0.02 ^a^	1.77 ± 0.01 ^b^	1.56 ± 0.04 ^c^	1.82 ± 0.03 ^a^
1028/1163	1.02 ± 0.10 ^a^	1.06 ± 0.09 ^a^	1.05 ± 0.11 ^a^	0.29 ± 0.01 ^b^	0.80 ± 0.09 ^c^
1743/2922	0.42 ± 0.03 ^a^	0.40 ± 0.02 ^a^	0.41 ± 0.02 ^a^	0.58 ± 0.03 ^b^	0.47 ± 0.03 ^c^
1743/2855	0.75 ± 0.04 ^a^	0.72 ± 0.04 ^a^	0.73 ± 0.04 ^a^	0.91 ± 0.06 ^b^	0.86 ± 0.09 ^b^

* The results are expressed as Average (±standard deviation); ^a–c^ Different letters in the same row indicate statistically different values (*p* < 0.05).

**Table 6:** Annotated metabolites in the coffee samples.

Metabolite	Chemical Group	Retention Time (min)	Precursor Ion (*m*/*z*)	MS/MS Fragments (*m*/*z*)
Benzoic acid *	Phenolic acids	4.53	121.1	121.5, 93.4, 77.5
Coumaric acid *	Phenolic acids	3.77	163.1	120.3, 93.4
Caffeic acid *	Phenolic acids	2.40	179.1	135.4, 107.3
Chlorogenic acid *	Phenolic acids	1.72	353.2	191.5
Quinic acid **	Organic acid	0.53	191.1	111, 173
Caffeoyl-quinolactone **	Hydroxycinnamate esters and lactones	1.31	335.1	161, 135, 179
p-Coumaroyl quinic acid **	Hydroxycinnamate esters and lactones	8.73	337.1	191, 163
Feruloyl-quinolactone **	Hydroxycinnamate esters and lactones	4.52	349.1	175, 193, 149, 134
Dicaffeoyl quinic acid **	Hydroxycinnamate esters and lactones	4.40	515.1	353, 335
Caffeoyl-feruloylquinic acid **	Hydroxycinnamate esters and lactones	5.12	529.1	367, 353
Dihydroxy-kaurenoic acid **	Diterpenes	6.40	333.2	303
Atracyligenin-*O*-hexoside **	Diterpenes	3.93	481.2	301
Isovaleryl-atractyligenin-*O*-hexoside derivative **	Diterpenes	6.41	565.3	481, 463, 303
Cafestol **	Diterpenes	7.75	315.1	285, 297, 267
Kahweol **	Diterpenes	7.54	313.1	283, 265, 295
Trihydroxy-octadecaenoic acid **	Fatty acids and derivatives	14.1	329.2	311, 293, 229, 171
Linoleic acid methyl ester **	Fatty acids and derivatives	7.22	293.2	236, 221
Caffeoyl-*N*-tryptophan **	Hydroxycinnamoyl amides	5.83	365.1	135, 229

* identification by analytical standards; ** identified by literature data.

**Table 7:** The docking scores of the examined phenolic acids and the co-crystallized inhibitors at the binding site of human acetylcholinesterase enzyme (PDB ID: 7XN1) and human alpha-glucosidase enzyme (PDB ID: 2QMJ).

Phenolic Acids	Human Acetylcholinesterase Enzyme (PDB ID: 7XN1)	Human Alpha-Glucosidase Enzyme (PDB ID: 2QMJ)
Docking Score (kcal mol^−1^)		
Tacrine	−8.64	NT ^1^
Acarbose	NT ^1^	−7.33
Benzoic acid	−6.02	−4.53
Caffeic acid	−6.00	−4.17
Chlorogenic acid	−7.25	−5.54
Coumaric acid	−5.72	−5.31

^1^ NT: Not Tested.

## Data Availability

The data presented in the study are available on request from the corresponding author.

## Supplementary Materials

The following supporting information can be downloaded at: https://www.mdpi.com/article/10.3390/antiox12061184/s1, Figure S1: Chromatographs and mass spectra of selected metabolites (a) quinic acid; (b) chlorogenic acid, (c) atracyligenin-O-hexoside, (d) feruloyl-quinolactone; Table S1: Mean contents of elucidated metabolites in coffee samples, expressed as normalized mean mass intensities.
