# Supplementary material for: Quality Assessment of Ground Coffee Samples from Greek Market Using Various Instrumental Analytical Methods, In Silico Studies and Chemometrics
Source: Antioxidants (Basel). 2023 May 30;12(6):1184. doi: 10.3390/antiox12061184 (PMC10295199; doi:10.3390/antiox12061184)
Supplement: Supplementary file 1 [file antioxidants-12-01184-s001.zip › antioxidants-2414455-supplementary.pdf]

antioxidants MDPI

Article

Table S1. Mean contents of elucidated metabolites in coffee samples, expressed as normalized mean mass intensities.

Metabolite	Coffees 101-125*	Coffees 201-211*	Coffees 301-306*	Coffees 401-406*	Coffees 501-509*
Benzoic acid	0.68(±0.28)[a]	0.60(±0.16)[a]	0.505(±0.062)a	0.581(±0.18)[a]	0.573(±0.076)[a]
Coumaric acid	0.46(±0.25)[a]	0.39(±0.31)[a]	0.50(±0.16)[a]	0.280(±0.043)[a]	0.26(±0.15)[a]
Caffeic acid	1.89(±0.48)[a]	1.73(±0.49)[a]	1.38(±0.63)[a]	1.485(±0.32)[a]	1.89(±0.73)[a]
Chlorogenic acid	10.2(±6.0)[a]	4.1(±1.3)[b]	2.56(±0.84)[b]	8.0(±3.2)[ab]	7.0(±3.0)[ab]
Quinic acid	40(±20)[a]	48(±14)[a]	42.4(±2.7)[a]	44.0(±8.0)[a]	41(±12)[a]
Caffeoyl-quinolactone	2.3(±2.0)[a]	3.1(±2.5)[a]	3.2(±2.8)[a]	0.97(±0.40)[a]	2.2(±2.0)[a]
p-Coumaroyl quinic acid	0.40(±0.56)[a]	1.0(±2.2)[a]	0.52(±0.35)[a]	0.39(±0.45)[a]	0.49(±0.43)[a]
Feruloyl-quinolactone	14.5(±9.0)[a]	11.6(±6.3)[a]	3.9(±2.5)[a]	17(±12)[a]	17(±10)[a]
Dicaffeoyl quinic acid	5.2(±7.8)[a]	2.9(±1.7)[a]	4.6(±5.4)[a]	5.3(±4.2)[a]	4.8(±6.1)[a]
Caffeoyl-feruloylquinic acid	3.9(±2.6)[a]	2.0(±1.5)[a]	1.43(±0.96)[a]	3.4(±2.5)[a]	3.2(±1.3)[a]
Dihydroxy-kaurenoic acid	3.4(±3.8)[a]	0	0	4.4(±6.6)[a]	0.27(±0.59)[a]
Atracyligenin-O-hexoside	47(±27)[a]	27(±19)[ab]	17(±15)[b]	21(±13)[ab]	26(±15)[ab]
Isovaleryl-atractyligenin-O-hexoside derivative	10.0(±6.3)[a]	9.1(±5.3)[a]	3.55(±0.97)[a]	5.0(±2.9)[a]	5.2(±2.9)[a]
Cafestol	1.1(±1.0)[a]	0.70(±0.82)[a]	0.161(±0.027)[a]	0.143(±0.025)[a]	0.92(±0.97)[a]
Kahweol	1.9(±1.2)[a]	1.81(±0.94)[a]	1.28(±0.76)[a]	1.00(±0.74)[a]	1.03(±0.68)[a]
Trihydroxy-octadecaenoic acid	10.2(±9.3)[a]	6.1(±6.7)[a]	3.6(±3.1)[a]	2.8(±1.7)[a]	8.6(±6.6)[a]
Linoleic acid methyl ester	0.40(±0.28)[a]	0.62(±0.53)[a]	0.63(±0.59)[a]	0.18(±0.16)[a]	0.41(±0.19)[a]
Caffeoyl-N-tryptophan	0.78(±0.89)[a]	1.17(±0.74)[a]	0.8(±1.8)[a]	0.91(±1.8)[a]	0.43(±0.62)[a]

[*] The results were expressed as Average(±standard deviation); [a-b] Different letters in the same row indicate statistically different values ($P < 0.05$).

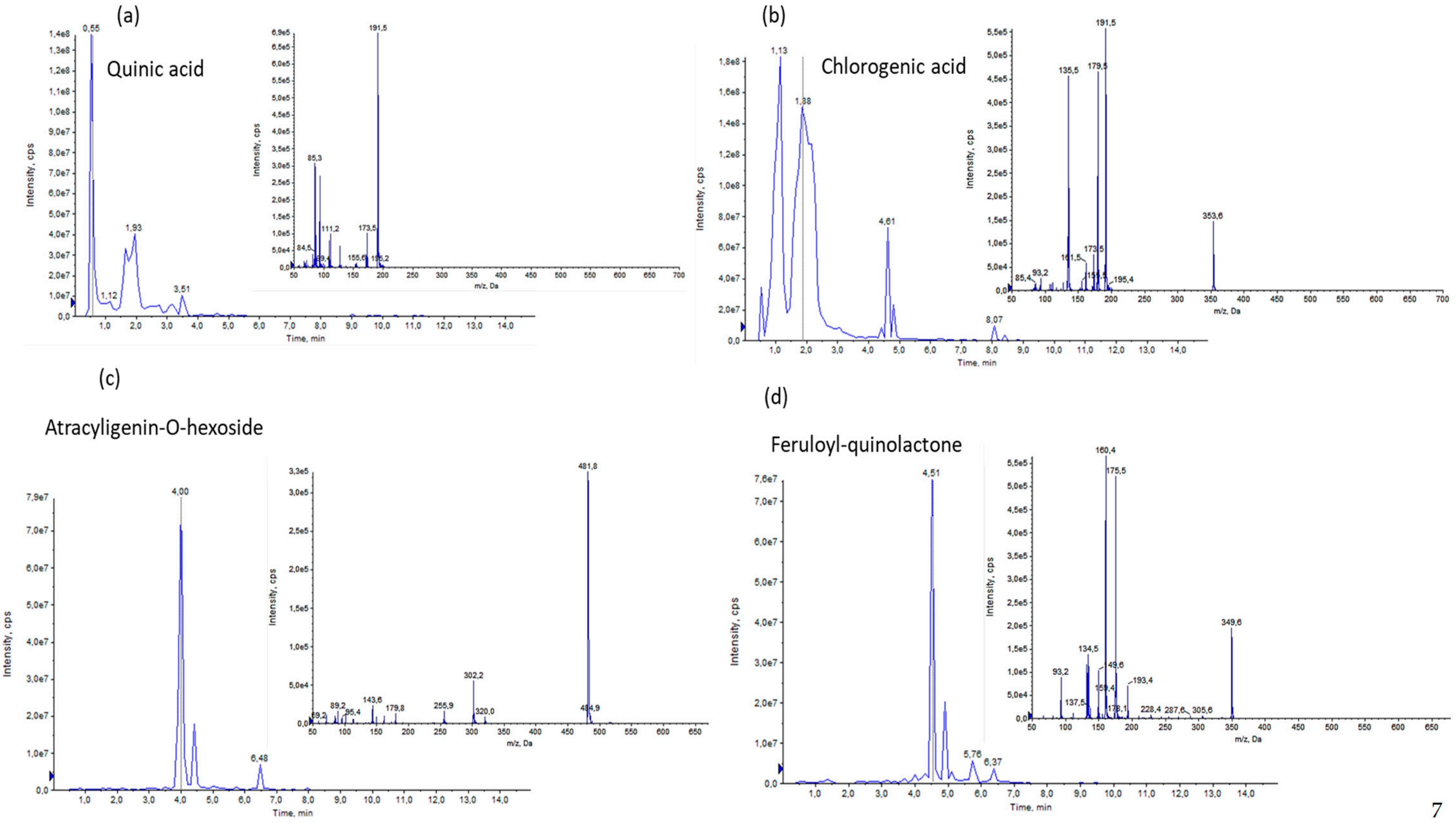

Figure S1. Chromatographs and mass spectra of selected metabolites **(a)** quinic acid; **(b)** chlorogenic acid, **(c)** atracyligenin-O-hexoside, **(d)** feruloyl-quinolactone.
